# Influenza virus neuraminidase regulates host CD8^+^ T-cell response in mice

**DOI:** 10.1038/s42003-020-01486-z

**Published:** 2020-12-08

**Authors:** Chung-Yi Wu, Hong-Yang Chuang, Chi-Huey Wong

**Affiliations:** 1 Genomics Research Center, Academia Sinica, 128 Academia Road, Sec. 2, Nankang Dist., Taipei, 115 Taiwan; 2 Department of Chemistry, The Scripps Research Institute, 10550N. Torrey Pines Rd., La Jolla, CA 92037 USA

**Keywords:** Glycoconjugates, Live attenuated vaccines

## Abstract

Influenza A virus (IAV)-specific CD8^+^ T-cell response was shown to provide protection against pandemic and seasonal influenza infections. However, the response was often relatively weak and the mechanism was unclear. Here, we show that the composition of IAV released from infected cells is regulated by the neuraminidase (NA) activity and the cells infected by NA-defective virus cause intracellular viral protein accumulation and cell death. In addition, after uptake of NA-defective viruses by dendritic cells (DCs), an expression of the major histocompatibility complex class I is induced to activate IAV-specific CD8^+^ T-cell response. When mice were infected by NA-defective IAV, a CD8^+^ T-cell response to the highly conserved viral antigens including PB1, NP, HA, M1, M2 and NS1 was observed along with the increasing expression of IL10, IL12 and IL27. Vaccination of mice with NA-defective H1N1 A/WSN/33 induced a strong IAV-specific CD8^+^ T cell response against H1N1, H3N2 and H5N1. This study reveals the role of NA in the IAV-specific CD8^+^ T-cell response and virion assembly process, and provides an alternative direction toward the development of universal influenza vaccines.

## Abstract (web summary)

Chung-Yi Wu et al. demonstrate a role of neuraminidase (NA) for Influenza A viruse (IAV)-specific CD8^+^ T-cell response and virion assembly. When mice are vaccinated with the live attenuated NA-defective IAV, they are protected from the upcoming IAV infection. This study provides insights into the development strategies of universal influenza vaccines.

## Introduction

Influenza A virus (IAV) has been a major threat to human health, causing seasonal outbreaks and pandemics with high morbidity and mortality especially involving more virulent strains such as 1918 A/H1N1, H5N1, and H7N9. Vaccination has been an effective strategy to control the spread of IAV, and the protective antibodies induced by the vaccine are known to recognize mainly the viral hemagglutinin (HA) domain and partially the NA moiety^1,2^. However, seasonal vaccines may lose their efficacy because of antigenic shift or drift in the circulating virus population through genomic reassortment or antigen mutations, respectively. Interestingly, certain populations, including pregnant women infected by some novel strains of IAV were asymptomatic with little antibody response, but had a steady IAV-specific CD8^+^ T-cell response to IAV-infected cells^1,2^. In addition, it was shown that humans, mice, and macaques with positive IAV-specific CD8^+^ T-cell response had cross-protective immunity against pandemic and seasonal influenza^3–7^, and the targets of CD8^+^ T-cell response were the highly conserved proteins from various IAV strains^8^. Despite the importance of CD8^+^ T-cell response in protection against influenza infection, the reported cellular immune response is often relatively weak and the mechanism has not been well understood.

In our previous study, we found that mice infected by a live attenuated IAV without the stalk or catalytic domain of NA were able to strongly induce the IAV-specific CD8^+^ instead of CD4^+^ T-cell response against the virus, leading to the development of a live attenuated influenza vaccine (LAIV)^4^. In this study, we further investigate the mechanism of such a selective immune response and elucidate the role of NA in the induction of IAV-specific CD8^+^ T cells against a broad range of IAV strains.

## Results

### NA activity regulated virus release

**Fig. 1:**
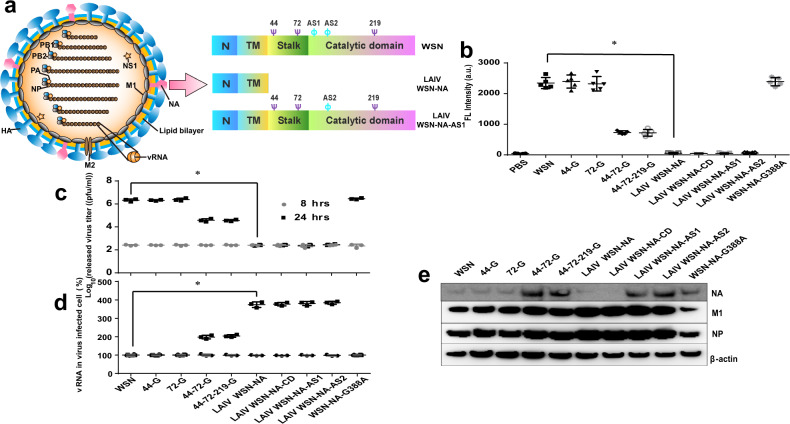
NA activity controls the release of virus. **a** Schematic overview of IAV and constructs of WSN and NA-defective WSNs: LAIV WSN-NA (deletion of the stalk and catalytic domains of NA) and LAIV WSN-NA-AS1 (inactivation of the active site of NA through R102A mutation). HA and NA are the two major surface glycoproteins of IAV and M2 is the third surface protein of IAV with ion channel activity. The matrix protein M1 is the most abundant protein to maintain viral structure. Viral RNA (vRNA) is the viral genome associated with the nucleoprotein (NP) to form the complex of vRNP. PB1, PB2, and PA are the components of RNA-dependent RNA polymerase. The NS1 protein is an interferon antagonist to facilitate viral replication. N, N-terminal cytoplasmic domain and cytoplasmic tails; TM, transmembrane domain. Purple Ψ, the glycosylation site; light blue ɸ, the active site of NA (AS). **b** NA activity was measured by the 4-MUNANA assay^4^. After A549 cells were infected with various viruses at an MOI of 3, the titers of viruses released from A549 cells (**c**) and the amount of vRNA (**d**) in A549 cells at 8 and 24-h post-infection were measured. **e** the total lysates from A549 cells were collected at 24-h post-infection by WSN and NA-defective WSNs (see Table 1) for analysis of the intracellular viral proteins HA, NP, and M1 by western blot. The filter was probed with anti-NA, anti-NP, anti-M1, and anti-β-actin antibodies. **b** Mean ± SD of five independent experiments. **c**, **d** Mean ± SD of three independent experiments. *P < 0.001.

We previously reported that immunization of mice with IAV without the stalk or catalytic domain of NA induced a strong IAV-specific CD8^+^ T-cell response with little antibody elicited^4^. In order to further understand the mechanism, WSN viruses with defective NA activity were generated by reverse genetics through deletion of the stalk or the catalytic domain (LAIV WSN-NA or LAIV WSN-NA-CD) or inactivation of the active site domain (LAIV WSN-NA-AS1 or LAIV WSN-NA-AS2) (Fig. 1a, b, Table 1). After A549 cells or MDCK cells were infected by defective NA viruses, only a small number of viruses was released in the supernatant compared to that from the cells infected by WSN with intact NA (Fig. 1c). In addition, the viral RNA (vRNA) (Fig. 1d) and viral proteins were accumulated inside the cells (Fig. 1e, Supplementary Fig. 7a), suggesting that the release of virus would require NA activity (Supplementary Figs. 1 and 7b).

**Table 1:** Characterization of different recombinant viruses.

Recombinant virus (name)	Distinguishing feature
**WSN**	**Wild type**
44-G	Deleted the glycosylation site 44 (N44A)
72-G	Deleted the glycosylation site 72 (N72A)
44-72-G	Deleted the glycosylation site 44 and 72 (N44A, N72A)
44-72-219-G	Deleted the glycosylation site 44, 72, and 219 (N44A, N72A, N219A)
**LAIV WSN-NA**	**Deleted the stalk and catalytic domain (S31stop)**
LAIV WSN-NA-CD	Deleted the catalytic domain (L75stop)
**LAIV WSN-NA-AS1**	**Deleted the active site domain 1 (N102A)**
LAIV WSN-NA-AS2	Deleted the active site domain 2 (N135A)
WSN-NA-G388A	Changed the amino acid residue 388G to A

WSN, LAIV WSN-NA, and WSN-NA-AS1 (bold words) are the major recombinant viruses used in this research.

### NA activity affected the virus-mediated host immune response

**Fig. 2:**
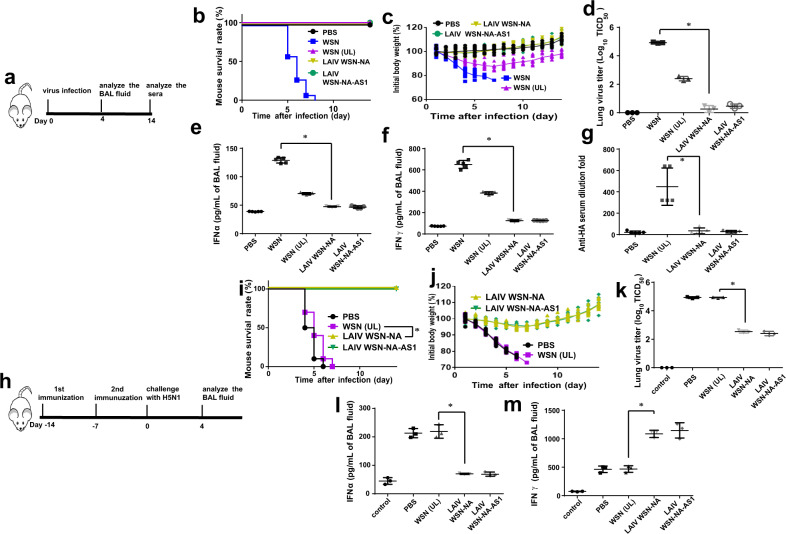
NA activity affected the host immune response in mice. **a** Schematic strategies used for mouse infection and analysis. After mice were infected with 1 × 10^6^ pfu of WSN, LAIV WSN-NA, LAIV WSN-NA-AS1 virus, or non-lethal dosage (1 × 10^3^ pfu) of WSN virus (WSN (UL)), the survival rate **b** and body weight **c** were recorded for 14 days. **d** IAV replication kinetics in the lungs of the virus-infected mice at day-4 post-infection. Measurement of IFNα **e** and IFNγ **f** from the BAL fluid of virus-infected mice at day-4 post-infection. **g** The sera from mice were analyzed using hemagglutination inhibition assay to measure the anti-HA antibody level at day-14 post-infection. **h** Schematic strategy used for mouse immunization and challenge. Analysis of survival rate **i** and body weight **j** of WSN, LAIV WSN-NA, LAIV WSN-NA-AS1, or PBS treated mice after challenge with a lethal dose of H5N1. **k** H5N1 virus replication kinetics in the lungs. IFNα **l** and IFNγ **m** from the BAL of the immunized mice as indicated at day-4 post-infection. The control indicated the group of mice without IAV infection. **b**, **i** Ten independent experiments are shown. **c**, **j** Mean ± SD of ten independent experiments. **d**, **e**, **f**, **g**, **k**, **l**, **m** Mean ± SD of five independent experiments. *P < 0.001.

To study whether the IAV without NA activity was the cause of reduced viral release and accumulation of viral proteins inside the infected cells leading to the biased host immune response^4^, we performed an animal study (Fig. 2a). Mice infected with the NA-defective virus (LAIV WSN-NA or LAIV WSN-NA-AS1) survived well and showed no body weight loss (Fig. 2b, c). The virus titers were lower in the lungs compared to non-vaccinated or WSN control groups with less interferon alpha and gamma (IFN-α/γ) induced in the bronchial alveolar lavage (BAL) fluid on day 4 after infection (Fig. 2d–f), and little anti-HA antibody was produced (Fig. 2g).

In the H5N1 challenge study (Fig. 2h), mice vaccinated with the NA-defective WSN also survived well without significant body weight loss (Fig. 2i, j) and were able to clear different strains of viruses from the lungs (Fig. 2k). Interestingly, the mice challenged with H5N1 virus induced less IFN-α (Fig. 2l) but more IFN-γ in BAL (Fig. 2m), indicating the activation of IAV-specific T-cell response. These results suggest that the activity of NA is an important factor in virulence, immune response, and probably is a key factor to be considered for the development of live attenuated influenza vaccines (LAIVs).

### NA-defective viruses, expression of MHC, and activation of IAV-specific CD8^+^ T cells

**Fig. 3:**
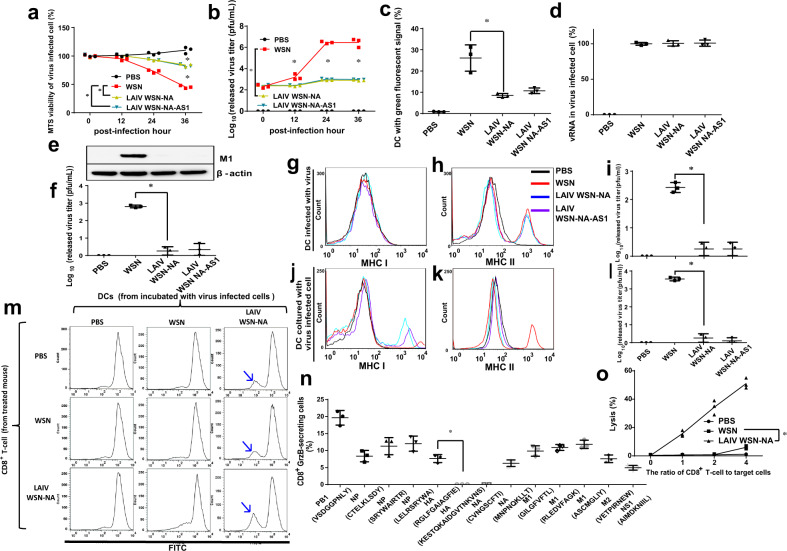
The NA activity regulated MHC I expression on DCs to prime and activate CD8^+^ T cells. **a** Analysis of the cell viability of virus-infected A549 cells by MTS assay. **b** Comparison of the amount of released virus at different time points from A549 cells. **c** Analysis of DCs with green fluorescent signal by flow cytometry after DCs were incubated with CFSE-labeled IAV-infected B cells in 24 h. % indicated the percentage of DCs with green fluorescent signal. 100% indicated 10,000 DCs. **d** Comparison of cellular vRNA at 6-h post-infection after B cells were infected with various viruses at an MOI of 3. **e** Analysis of viral protein M1 in DCs by western blot. The filter was probed with anti-M1 and anti-β-actin monoclonal antibodies. **f** Virus released from DCs after incubation with CFSE-labeled B cells infected by virus. Analysis of MHC I **g** and MHC II **h** expression by flow cytometry of DCs, and virus replication in IAV-infected DCs (**i**). Some isolated DCs were incubated in DC culture medium for another 24 h at 37 °C for detection of virus replication. **i** Representative flow cytometry histograms of MHC I **j** and MHC II **k** expression on DCs after incubation with IAV-infected B cells. **l** Virus released from DCs after incubation with IAV-infected cells. **m** Representative flow cytometry histograms of CD8^+^ T-cell proliferation. **n** Distribution of IAV proteins recognized by the CD8^+^ T cells induced by NA-defective viruses. The epitope regions of viral proteins were shown in the figure. The peptides were incubated with CD8^+^ T cells and GrzB-secreted cells and measured by flow cytometry. **o** Cytotoxicity of IAV-specific CD8^+^ T cells. **a**, **b**, **c**, **d**, **f**, **i**, **l**, **n**, **o** Mean ± SD for three independent experiments. **g**, **h**, **j**, **k**, **m** Data are representative of duplicate experiments. *P < 0.001.

To understand how responses to NA-defective viruses might activate IAV-specific CD8^+^ T cells, we first investigated the interaction of dendritic cells (DCs) with IAVs and IAV-infected cells to understand how they were processed by DCs for presentation to T cells and other immune cells^9,10^. After A549 cells were infected by NA-defective WSN viruses, the cells showed increased viability (Fig. 3a) and released fewer viruses into the supernatant as compared to the WSN-infected cells (Fig. 3b). It was also found that the distribution of some viral proteins inside the cell was not significantly affected, probably due to the different processes of cell death in different IAV-infected cells and viral proteins accumulated in NA-defective virus-infected cells (Supplementary Fig. 2). The green fluorescent signals were relatively weak compared to WSN (Fig. 3c) in DCs co-cultured with mouse B cells labeled with 5(6)-carboxyfluorescein diacetate N-succinimidyl ester (CFSE) and infected with NA-defective WSN that had similar vRNA levels at 6-h post-infection to WSN-infected B cells (Fig. 3d), suggesting that the NA activity might affect the uptake of IAV antigens by DCs. When DCs were cultured with the cells infected by WSN, more DCs with green fluorescent signal and more viral protein M1 inside the DCs (Fig. 3e, Supplementary Fig. 7c) were observed. After further incubation at 37 °C for 24 h, more viruses were released to the supernatant (Fig. 3f), suggesting that WT viruses replicated in DCs.

To further study the effects of DCs infected by IAV, the major histocompatibility complex class I (MHC I) and class II (MHC II), which are essential for presentation of the internalized molecules after processing, were measured by flow cytometry^9^. In general, we observed that when DCs were infected by WSN or co-cultured with the cells infected by WSN, MHC II was more expressed than MHC I (Fig. 3g, h, j, k), and the virus replicated more in DCs (Fig. 3i). However, when DCs were co-cultured with the cells infected by NA-defective WSN viruses, MHC I was more expressed whereas MHC II was not expressed on DCs (Fig. 3j, k) and little virus was released to the medium (Fig. 3l). We also found that when DCs were incubated with inactivated IAVs, more MHC II than MHC I was expressed (Supplementary Fig. 3a, b); however, when DCs were incubated with the cells treated with the inactivated virus, the expression of MHC I/II was not increased (Supplementary Fig. 3c, d).

MHC I-expressed DCs can prime and activate the proliferation of CD8^+^ T cells and secretion of IFN-γ^9^. LAIV WSN-NA treated mice would induce IAV-specific CD8^+^ T cells^4^. When DCs were co-cultured with the B cells infected by NA-defective viruses, the isolated DCs were incubated with CD8^+^ T cells from mice treated with different IAVs. As expected, the DCs induced a proliferation of CD8^+^ T cells (Fig. 3m) accompanied with IFN-γ secretion (Supplementary Fig. 4a), especially when DCs were incubated with the CD8^+^ T cells isolated from the mice vaccinated with LAIV WSN-NA produced from MDCK cells. This result suggested that the uptake of antigens by DCs from the cells infected by NA-defective viruses would prime and activate the IAV-specific CD8^+^ T cells.

The IAV-specific CD8^+^ T cells induced by NA-defective WSN were able to recognize the epitopes from different IAV proteins such as PB1, NP, HA, M1, M2, and NS1, as shown by their secretion of granzyme B (GrzB) by flow cytometry (Fig. 3n). In addition, the CD8^+^ T cells were more activated when incubated with different combinations of viral peptides (Supplementary Fig. 4b). Furthermore, when the cells infected by NA-defective WSN were incubated with different ratios of CD8^+^ T cells, the lysis of the IAV-infected cells (Fig. 3o) and the secretion of IFN-γ (Supplementary Fig. 4c) showed a dose-dependent response, suggesting that the NA-defective virus could induce an immune response in which CD8^+^ T cells recognize and eliminate the cells infected by IAV. The observation that vaccination of mice with LAIV WSN-NA elicited a broadly protective immune response against different strains and subtypes of IAVs, including WSN, A/Puerto Rico/8/1934 (H1N1), A/Solomon Islands/3/2006 (H1N1), A/New Caledonia/20/1999 (H1N1), A/California/07/2009 (H1N1), A/Brisbane/10/2007 (H3N2), and H5N1 in the challenge study suggests that LAIV WSN-NA induced IAV-specific CD8^+^ T cells have the ability to recognize many conserved viral antigens (Supplementary Fig. 5).

### NA-defective viruses and expression of cytokines in host immune response

**Fig. 4:**
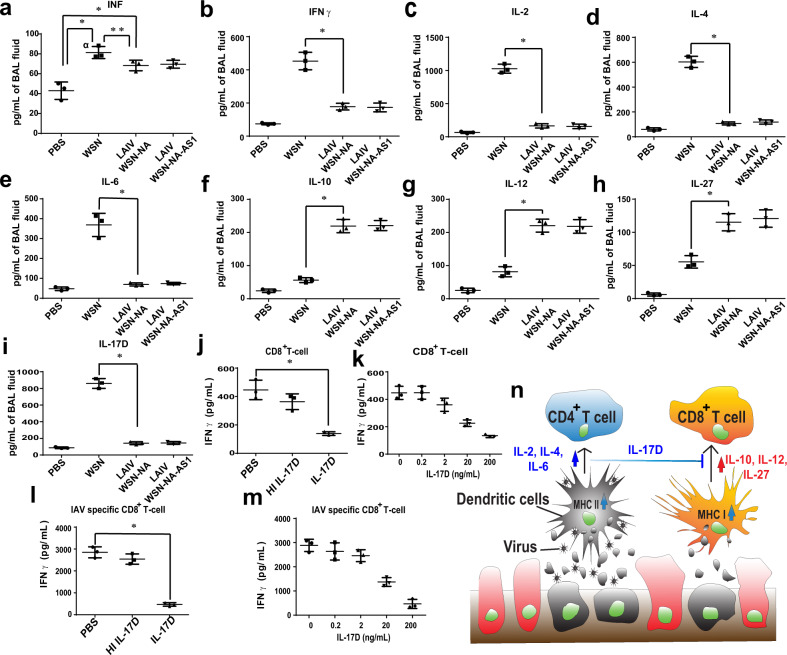
The NA activity affects cytokines production. **a** INFα, **b** IFNγ, **c** IL-2, **d** IL-4, **e** IL-6, **f** IL-10, **g** IL-12, **h** IL-27, and **i** IL-17D production from the mice infected with 1 × 10^3^ pfu of IAV at 4-day post-infection. **j** IL-17D inhibited the induction of IAV-specific CD8^+^ T cells. HI IL-17D: Heat inactivated IL-17D. **k** The relationship between the dose of IL-17D and the secreted IFNγ from CD8^+^ T cells. **l** IL-17D suppressed the activation of IAV-specific CD8^+^ T cells. **m**` Dose-dependent suppression of IFNγ secreted from the IAV-specific CD8^+^ T cells by IL-17D. **n** A model of IAV-specific CD8^+^ T-cell induction and activation. CD8^+^ T-cell induction was suppressed by IL-17D but activated by IL-27. The gray cell indicated IAV-infected cell. **a**–**m** Mean ± SD for three independent experiments. *P < 0.001, **P < 0.05.

The mechanism of immune response to IAV infection that causes tissue injury in the host is complicated, and it is known that the cellular activities of innate and adaptive immune response can be affected by cytokines^11,12^. Here we found that mice infected by WSN induced higher levels of IFNα, IFNγ, IL-2, IL-4, and IL-6 known to activate the antibody response, while the NA-defective WSN induced higher levels of IL-10, IL-12, and IL-27 known to trigger the cellular immune response (Fig. 4a–h)^13–18^. In a recent study, IL-17D was shown to be a critical cytokine during IAV infection to suppress the activity of CD8^+^ T cells through regulation of dendritic cells^19^. We also found that mice immunized with NA-defective WSN induced a very low level of IL-17D (Fig. 4i), likely minimizing its suppression of the CD8^+^ T-cell induction (Fig. 4j, k) and activation (Fig. 4l, m). This result was supported by the observation that IL-17D was able to reduce the secretion of INFγ from CD8^+^ T cells (Fig. 4j, k) or IAV-specific CD8^+^ T cells (Fig. 4l, m)^19^. We speculate that when IAV was taken by DCs, MHC II, and the cytokines including IL-2, IL-4, and IL-6 were expressed to activate CD4^+^ T cells, and IL-17D was induced to suppress the CD8^+^ T-cell activity. However, the expression of MHC I on DCs to prime the IAV-specific CD8^+^ T cells required the uptake of IAV-infected cells and the cooperation of cytokines such as IL-10, IL-12, and IL-27 (Fig. 4n).

**Fig. 5:**
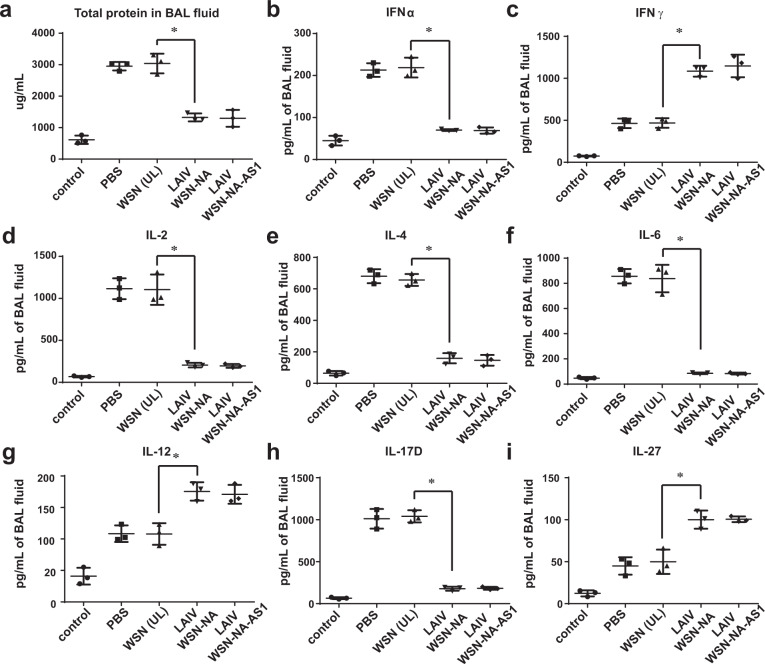
NA activity affects the profile of cytokine expression in host defense. **a** Total protein, **b** INFα, **c** IFNγ, **d** IL-2, **e** IL-4, **f** IL-6, **g** IL-12, **h** IL-17D, and **i** IL-27 production from mice immunized with different viruses at 4-day post-infection in a challenge study with H5N1. The control indicated the group of mice without IAV infection. **a**–**i** Mean ± SD for three independent experiments. **P < 0.05. *P < 0.001.

Severe influenza infection causes a cytokine storm that originates from the hyperinduction of proinflammatory cytokine production^20,21^. Interestingly, the mice with robust IAV-specific CD8^+^ T-cell activation could avoid the cytokine storm caused by highly pathogenic IAV infection. When mice were vaccinated with LAIV WSN-NA followed by infection with a high lethal dosage of H5N1, the mice produced less total protein in the BAL fluid and lower levels of IFN-α, IL-2, IL-4, IL-6, and IL-17D, but higher levels of IFNγ, IL-12, and IL-27 to activate the IAV-specific CD8^+^ T-cell response, suggesting that the NA activity would affect the course of host immune response to IAV infection (Fig. 5).

### NA activity and composition of viruses

**Fig. 6:**
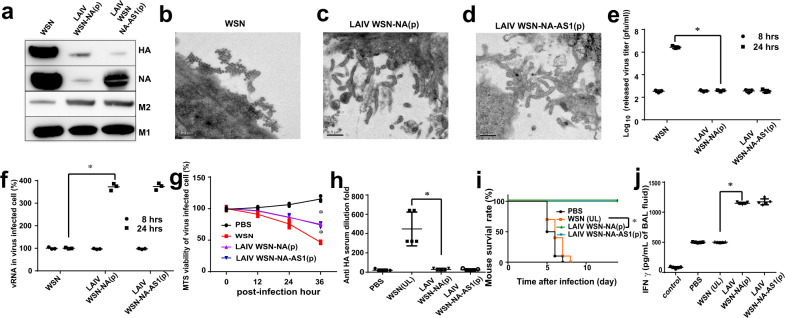
Characterization of the released viral particles from MDCK cells infected with NA-defective viruses. **a** Analysis of major proteins in the NA-deffective viral particles by western blot. The filter was probed with anti-HA, anti-NA, anti-M2, and anti-M1 monoclonal antibodies. Morphology of WT virus (**b**), LAIV WSN-NA(p) (**c**), and LAIV WSN-NA-AS1(p) (**d**) examined by transmission electron microscopy. **e** Comparison of the viral particles released from LAIV WSN-NA(p) and WSN-NA-AS1(p) infected A549 cells, and **f** vRNA in A549 cells infected with the NA-deffective viral particles at 24-h post-infection. **g** Cell viability of A549 cells infected with the NA-deffective viral particles. **h** The sera of immunized mice were analyzed using hemagglutination inhibition assay to measure the anti-HA antibody level. **i** Analysis of the survival rate of mice immunized with WSN, LAIV WSN-NA(p), WSN-NA-AS1(p), or PBS after challenge with H5N1. **j** Following (**i**), IFNγfrom BAL fluid was measured at day-4 post-infection. The control indicated the group of mice without IAV infection. **e**, **f**, **g** Mean ± SD of three independent experiments. **h**, **j** Mean ± SD of five independent experiments. **i** Ten independent experiments are shown. *P < 0.001.

Unexpectedly, we also found that the composition of released viruses from IAV-infected cells was affected by NA-defective strains. At the late stage of NA-defective IAV infection in MDCK cells (on 18-h post-infection), the cells released some particles (LAIV WSN-NA(p) and LAIV WSN-NA-AS1(p)) that had different distributions of proteins (Supplementary Fig. 6a). These particles contained more M2 (ion channel) protein and less HA protein (Fig. 6a, Supplementary Fig. 7d), but still had the viral genome (vRNA) (Supplementary Figs. 6b, 7e) albeit with lower HA-sialic acid binding ability (Supplementary Fig. 6c), and were mainly elongated, filamentous, and irregular-shaped particles (Fig. 6b–d).

### Application to universal vaccine design

After A549 cells were infected by LAIV WSN-NA(p) and LAIV WSN-NA-AS1(p) produced from MDCK cells, there were fewer viruses released into the supernatant (Fig. 6e), and the intracellular viral genome (Fig. 6f) and protein M1 (Supplementary Figs. 6d, 7f) were accumulated, along with a reduction in viable cells (Fig. 6g). When mice were infected with 1 × 10^6^ pfu of LAIV WSN-NA(p) or LAIV WSN-NA-AS1(p) via oral administration, they survived well with no body weight loss, and were able to effectively clear the infection particles (Supplementary Fig. 6e–g). In addition, the mice induced less IFN-α/γ in the BAL fluid (Supplementary Fig. 6h, i) and produced very low levels of anti-HA antibodies (Fig. 6h), indicating that these particles could be used as effective LAIVs with low pathogenicity. In the H5N1 challenge study, the immunized mice were able to survive well without body weight loss (Fig. 6i, Supplementary Fig. 6j), clear the virus from the lungs (Supplementary Fig. 6k), and induce less IFN-α (Supplementary Fig. 6l) and more IFN-γ (Fig. 6j), suggesting that the IAV-specific CD8^+^ T-cell response was activated. Since the immune responses of LAIV WSN-NA(p) and LAIV WSN-NA-AS1(p) were similar to that of NA-defective WSN, we concl uded that the induction of IAV-specific CD8^+^ T-cell response does not require the release of much infective virus, further supporting the important role of NA in the process of immune response.

## Discussion

It is known that infection with one subtype of IAV may induce IAV-specific CD8^+^ T cells to target different subtypes of IAV^6,8,22^, or IAV-specific CD4^+^ T cells to target that specific subtype of IAV^8,23^. However, patients with severe IAV infection usually have a limited or severely compromised CD8^+^ T-cell proliferation and differentiation response to eliminate the virus^6,24,25^. Our study indicated that though CD4^+^ T cells were generally induced in response to IAV infection, and IL-17D was expressed to suppress the CD8^+^ T-cell activity, MHC I was expressed to activate CD8^+^ T cells in the presence of NA-defective IAV. In addition, the mice infected with WT IAV did not generate higher CD8^+^ T cells through induction of cytokines such as IL-10, IL-12, and IL-27^18,26^.

Recent studies have suggested strategies for the design of LAIV to elicit CD8^+^ T cells for cross protection^4,27–30^, and uptake of large amounts of target antigens by DCs will stimulate an efficient activation of CD8^+^ T cells^31–34^. However, our study showed that the viral particles released from MDCK cells infected by NA-defective WSN would provide sufficient antigens for DCs to prime the IAV-specific CD8^+^ T cells and eliminate IAV-infected cells through recognition of conserved cytotoxic T lymphocyte epitopes of various IAV antigens such as PB1, NP, HA, M1, and NS1.

During the process of influenza infection, the cytoplasmic tails of HA and NA play an important role in the budding process. The concentration of HA and NA in lipid raft provides a local alteration in membrane curvature resulting in the interaction of the cytoplasmic tails of HA and NA with M1 protein to start the budding process. M1 recruits M2 to the budding site for membrane scission and NA then cleaves the sialic acid linkage on the cell surface to release the budding virion^35–38^. In our study, the NA-defective viruses can still express the cytoplasmic tails of NA and produce infection particles in MDCK cells but not in A549 cells, probably because the MDCK cell is highly polarized and susceptible for IAV replication^39^. Surprisingly, these infection particles have different compositions, especially with increase in M2 and decrease in HA expression, suggesting that NA is used not only for the cleavage of sialic acid ligands to release virions but its ectodomain also affects virion composition in the budding process.

In summary, this study has demonstrated that the NA-defective WSN or the viral particles produced from MDCK cells infected with NA-defective WSN can be used as LAIV to effectively induce IAV-specific CD8^+^ T-cell response to protect against different influenza strains such as H1N1, H3N2, and H5N1. The LAIV particles can be easily produced and this approach may lead to a new direction toward the development of a universal influenza vaccine.

## Methods

### Cell lines and viruses

Madin-Darby canine kidney cells (MDCK) and human embryonic kidney cells (HEK293T) were maintained in Dulbecco’s modified Eagle’s medium (DMEM) (Invitrogen, Rockville. MD). A549 human adenocarcinoma alveolar basal epithelial cells were kept in F-12K medium (Invitrogen, Rockville, MD). All media were supplemented with 10% heat-inactivated fetal bovine serum (FBS) (Thermo Scientific) and antibiotics (100 U/ml penicillin G and 100 gm/ml streptomycin). The MDCK cells with stable NA expression were prepared by cloning the full-length NA (from WSN strain) into a cDNA expression lentivector (pLAS2w.Ppuro) to generate NA-expression lentivirus using the protocol provided by the National RNAi Core Facility, Academia Sinica, Taiwan (rnai.genmed.sinica.edu.tw) and were maintained in DMEM medium^4^. Influenza A virus, A/WSN/33 strain was used in the studies.

### Antibodies

Mouse monoclonal anti-HA, anti-NA, and anti-NP antibodies were obtained from Sino Biological. Mouse monoclonal anti-β-actin and rabbit monoclonal anti-MHCII antibodies were purchased from Millipore. Goat polyclonal anti-M1 was purchased from Santa Cruz Biotechnology. Mouse monoclonal anti-M2 antibody was obtained from ABcan. Rabbit polyclonal anti-granzyme B antibody was purchased from Aviva Systems Biology. Rabbit monoclonal anti-MHC I antibody was obtained from Invitrogen. All commercial antibodies were validated for specificity by companies and us via western blot.

### Virus replication rate

Monolayer cultures of MDCK and A549 cells in 12-well dishes were washed twice with 1× phosphate-buffered saline (PBS). The cells were infected with variants of modified influenza virus at an MOI of 3 in serum-free medium containing 0.1 g/mL L-(tosylamido-2-phenylethyl) chloromethyl ketone (TPCK)-trypsin (Pierce) and incubated at 37 °C for 1 h; cells were washed twice with 1x PBS and then incubated with complete medium. At different time points, the supernatant was collected to determine the virus titer by plaque assay in MDCK cells^36^.

### Plaque assay

Monolayers of MDCK cells (for WSN) or MDCK cells with stable NA expression (for NA-defective WSN such as LAIV WSN-NA, LAIV WSN-NA-AS1, LAIV WSN-NA(p), and LAIV WSN-NA-AS1(p)) in 6-well dishes were washed twice with 1x PBS. The cells were then inoculated with serial 10-fold dilutions of the virus in serum-free medium containing 0.5 μg/mL TPCK-trypsin and incubated at 37 °C for 1 h. Afterward, the cells were washed and overlaid with MEM containing 0.5% agarose (Lonza) and 0.5 gm/ml TPCK-trypsin, and after 3 days, fixed with 10% formaldehyde and stained with 0.1% crystal violet solution^4^.

### Generation of recombinant viruses

Eight fragments of A/WSN/33 viral genome were amplified by RT-PCR. As shown in Table 1, we mutated the N residue to A in the putative sequon N-X-S/T to delete the glycosites 44, 72, and 219 (i.e., N44A mutation designated as 44-G), or added a stop codon to the first amino acid residue of the stalk or catalytic domain in the NA genome to remove the stalk and/or catalytic domain (designated as LAIV WSN-NA (S31stop) and LAIV WSN-NA-CD (L75stop)) of NA as previously described^4^, or changed the designated amino acid in the active site (AS1: R102A (designated as LAIV WSN-NA-AS1); AS2: D135A (designated as LAIV WSN-NA-AS2)) by site-directed mutagenesis. The viral cDNAs were inserted into pcDNA3.1 containing the pol I and CMV promoter with the method similar to the way pHW2000 was generated. The recombinant viruses were generated by the 8-plasmid co-transfection method into MDCK/293T cells with stable NA expression following the method described previously by our group^4^. Supernatants were collected, titrated, and frozen at −80 °C until use.

### RNA isolation, reverse transcription, and quantitative PCR

The total intracellular or virus particle RNA was extracted by using Trizol (Invitrogen) and EasyPrep total RNA kit (TOOLS, Taiwan) according to the manufacturer’s protocols. The reverse transcription (RT) reaction was carried out using the SuperScript III first-strand synthesis system (Invitrogen). To synthesize the cDNA of vRNA, the influenza A-specific primer (uni-12; 5′-AGCAAAAGCAGG-3′) was used. The primer with the sequence 5′-AGGTCCAGACGCAGGATGGC-3′ was used for β-actin in the RT reaction^36^. To quantify vRNA, quantitative PCR was performed by using SYBR green 1 (Roche, Germany). To detect influenza vRNA, the primers used were 5′-AATAAGACGAATCTGGCGCCAAGC-3′ and 5′-CAGCCGTTGCATCGTCACCA-3′. To quantify β-actin, the primers used were 5′-GCAAGCAGGAGTATGACGAGTCCG-3′ and 5′-GCATTTGCGGTGGACGATGG-3′. To quantify the total intracellular and cytoplasmic vRNA of influenza A virus, the threshold cycle (CT) values of vRNA were normalized to β-actin^36^. For amplification of influenza A NP, HA, NA, and M segments, the primer sets for NP were 5′-ATGGCGACCAAAGGCACCAAAC-3′ and 5′-CTTTGTCATAAAGGATGAGTTC-3′, for HA were 5′-ACAATATGTATAGGCTACCATG-3′ and 5′-TCTGTATTGAATGGATGGGATG-3′, for NA were 5′-ATGAATCCAAACCAGAAAATAATAAC-3′ and 5′-AGATGAATTGCCGGTTAATATC-3′, and for M were 5′-ATGAGTCTTCTAACCGAG-3′ and 5′-ACTGGCAAGTGCACCAGCAG-3′^36,40^.

### Preparation of WSN and NA-defective WSN viruses for immunogenicity test

WSN, LAIV WSN-NA(p) and LAIV WSN-NA-AS1(p) were cultured in MDCK cells, and LAIV WSN-NA and LAIV WSN-NA-AS1 viruses were cultured in MDCK cells with stable expression of NA. For the preparation of inactivated viruses, viruses were inactivated by using 0.1% BPL (Acros Organics, Geel, Belgium) at room temperature for 24 h followed by dialysis for 24 h against PBS buffer. Inactivation of virus was confirmed by performing culture on MDCK cells^4^. For analysis of the virulence of recombinant virus, each group of ten female 4–6-week-old BALB/c mice was intranasally inoculated with 50 μL of virus (1 × 10^3^ pfu for WSN (UL) and 1 × 10^6^ pfu for WSN, LAIV WSN-NA, LAIV WSN-NA-AS1, LAIV WSN-NA(p), or LAIV WSN-NA-AS1(p)). The survival rate and body weight changes were recorded daily for 14 days post-infection. For the immunogenicity test, mice were immunized twice intranasally with 1 × 10^3^ pfu of WSN (UL), or with 1 × 10^6^ pfu of LAIV WSN-NA, LAIV WSN-NA-AS1, LAIV WSN-NA(p), or LAIV WSN-NA-AS1(p) on days 0 and 21. One week after the booster immunization, mice were anesthetized and inoculated intranasally with 10xLD_50_ of H5N1 virus and separated into three groups. In the first group, the survival rate and body weight changes were recorded daily for 14 days post-infection. In the second group, the BAL fluids were collected to measure the production of IFN-α/γ, IL-2, IL-4, IL-6, IL-10, IL-12, IL-17D, and IL-27, and the extent of IAV replication; and in the third group, the serum sample was collected for analysis of antiserum production. For the challenge assay, the immunized mice were inoculated intranasally with 10 × LD_50_ of WSN, A/Puerto Rico/8/1934 (H1N1), A/Solomon Islands/3/2006 (H1N1), A/New Caledonia/20/1999 (H1N1), A/California/07/2009 (H1N1), A/Brisbane/10/2007 (H3N2), and H5N1, and separated into two groups. In the first group, the survival rate and body weight changes were recorded daily for 14 days after the infection. In the second group, the BAL fluids were collected to measure IAV replication. All animal experiments were evaluated and approved by the Institutional Animal Care and Use Committee of Academia Sinica.

### Hemagglutination inhibition assay

After serum samples were serially diluted twofold in a 96-well plate, 4 hemagglutination units (HAU) of WT WSN were added to each well for 1 h at room temperature. After incubation, 25 µL of a 2% (vol/vol) turkey erythrocyte solution was added to give a total volume of 125 µL and the mixture was incubated for 1 h at room temperature. The HAI titer of individual serum samples was determined to be the inverse of last dilution where cells were not agglutinated^4^.

### Measurement of IFN-α/γ and other cytokines

IFN-α/γ, IL-2, IL-4, IL-6, IL-10, IL-12, IL-17D, and IL-27 were measured by using ELISA kit according to the manufacturer’s protocol (IFN-α: Cloud-Clone Corp; IFN-γ: Boster Biological Technology Co., Ltd: IL-2, IL-4, IL-6, IL-10, IL-12, IL-17D, and IL-27: R&D Systems).

### Immunofluorescence microscopy

MDCK cells grown in slide chambers were infected by WSN, LAIV WSN-NA, or LAIV WSN-NA-AS1 at an MOI of 3. After 24 h, the cells were fixed with 4% paraformaldehyde and permeabilized with 0.5% saponin. After being blocked with 0.25% bovine serum albumin, the cells were incubated with mouse monoclonal anti-HA, anti-NP, anti-M2, and anti-M1 antibodies (ABcam). After extensive washes, the cells were stained with goat anti-mouse secondary antibody conjugated to Alexa Fluor 488 (ABcam). Last, images were captured with an inverted fluorescence microscope (Leica DMI6000).

### Uptake of antigens from IAV-infected cells by DCs

B cells from normal mice were infected with LAIV or incubated with inactivated WSN (BPL treatment) at an MOI of 1 in RPMI 1640 medium containing 0.5 μg/ml TPCK-trypsin and incubated at 37 °C for 1 h. The infected cells were washed five times with PBS and labeled with CFSE (Merck) (1 μM CFSE for 10 min at room temperature, then washed twice with complete RPMI 1640 plus 10% FCS), then incubated with GM-CSF-cultured bone marrow-derived dendritic cells (BMDC) (ratio: 1:3) in DC culture medium (RPMI 1640 supplemented with 20 ng/mL murine GM-CSF (R&D Systems), 10% FBS, 50 μM 2-ME, 100 units/mL penicillin, and 100 μg/mL streptomycin) for 24 h at 37 °C. After incubation, DCs were isolated by M-pluriBead Cell Separation kit (pluriSelect) following the procedure from the company. The number of DCs with the green fluorescent signal was analyzed by flow cytometry, and viral proteins were analyzed by western blot^41^. Some isolated DCs were incubated in DC culture medium for another 24 h at 37 °C for detection of virus replication.

### MHC I and MHC II expression on DCs infected by IAV or co-cultured with IAV-infected cells

DCs were infected with live WSN or incubated with inactivated WSN (BPL treatment) at an MOI of 3 in RPMI 1640 medium containing 0.5 μg/ml TPCK-trypsin and incubated at 37 °C for 1 h. Then, the DC cells were washed twice and incubated in DC culture medium for 24 h at 37 °C, then analyzed for MHC I and MHC II expression by flow cytometry. For the study of DCs interacting with IAV-infected cells, IAV-infected B cells (as described previously) were co-cultured with DCs (ratio: 3:1) in DC culture medium for 24 h at 37 °C. Then, DCs were isolated by using M-pluriBead Cell Separation kit (pluriSelect) and analyzed to measure the MHC I and MHC II expression by flow cytometry.

### IAV-infected cells regulate DCs to affect the proliferation of CD8^+^ T cells

After DCs were incubated with the B cells infected by WSN or LAIV WSN-NA virus, DCs were isolated and co-cultured with CFSE-labeled CD8^+^ T cells (1 μM CFSE for 10 min at room temperature, then washed twice with complete RPMI 1640) (ratio: 1:2) from mice treated with PBS, WSN, or LAIV WSN-NA in complete RPMI 1640 with 0.5 ng/mL IL-7 (R&D Systems), 30 U/mL IL-2 (R&D Systems), and 50 μM 2-ME for 24 h at 37 °C. CD8^+^ T cells were then isolated by Dynabeads Untouched Mouse CD8 Cells kit (Invitrogen) following the procedure from the company and the number of cells with FITC signal was analyzed by flow cytometry.

### Suppression of CD8^+^ T-cell activity by IL-17D

After DCs incubated with the B cells infected by LAIV WSN-NA virus, the DCs were isolated and co-cultured with CD8^+^ T cells (from PBS or LAIV WSN-NA treated mice) (ratio: 1:2) and IL-17D (0–200 ng/mL, R&D Systems). After 3 days, the supernatants were collected to measure the IFN-γ production by ELISA kit. Heat inactivated IL-17D was prepared by heating at 56 °C for 30 min.

### CD8^+^ T cytotoxicity assay

CD8^+^ T cells were isolated from the mice immunized with WSN, LAIV WSN-NA, or PBS using Dynabeads Untouched Mouse CD8 Cells kit (Invitrogen) and co-cultured with CFSE-labeled B cells infected with LAIV WSN-NA at an MOI of 3 in complete RPMI 1640. The co-cultures were run at different cell ratios (i.e., 1:1, 2:1, and 4:1 CD8^+^ T cell/IAV-infected cell) in 200 μl of RPMI 20% in U-bottom 96-well plates. Afterward, the mortality of IAV-infected cells was scored by flow cytometry. The value of cell mortality was normalized to that of IAV-infected cells without co-culturing with CD8^+^ T cells as IAV-infected cells would cause cell apoptosis.

### Flow cytometry

Cells were harvested and suspended in FACS buffer [2% (vol/vol) FBS in PBS] at a density of 10^6^/ml. The antibody used in this study was anti-MHC I, anti-MHC II, or anti-granzyme B antibody. Cellular fluorescence intensity was analyzed by FACS Canto (BD Biosciences) and FCS Express 3.0 software.

### Cell-binding assay

Turkey erythrocytes were pretreated with different amounts (0–60 μg/mL) of Vibrio cholerae neuraminidase [receptor destroying enzyme (RDE)] (Sigma) for 60 min at 37 °C, then washed once with PBS and divided into 2% (vol/vol) erythrocyte solutions in PBS. Twenty-five microliters of each 2% solution were added to a solution of virus containing the same amount of M1 protein (determined by western blot) to give a total volume of 125 μL. The virus and RDE-treated erythrocytes were incubated for 1 h at room temperature, and agglutination was then measured. Data were expressed as the maximal concentration of RDE that gave full agglutination^4^.

### Transmission electron microscopy

MDCK cells were grown on ACLAR embedding film with 7.8-mil thickness (E, M, S) for 1 day followed by infection with influenza virus at an MOI of 5. At 24 hpi, the virus-infected cells were rinsed with 0.1 M cacodylate buffer and fixed with 2.5% glutaraldehyde in 0.1 M cacodylate buffer at 4 °C for 30 min. Then cells were postfixed with 1% osmium tetroxide in 0.1 M cacodylate for 30 min, stained with 1% uranyl acetate and lead citrate for 1 h, dehydrated with ethanol, and embedded with resin. After baking, the sample was cut into 80-nanometer thin sections using ultramicrotome and examined with Tecnai G2 Spirit TWIN (FEI Company)^36^.

### Statistics and reproducibility

All data were presented as means ± standard error of the mean. The numbers of sample and replicates of experiments were shown as mentioned in the figure legends. Comparisons between groups were determined using Student’s *t* test. Differences were considered significant at *P < 0.001, **P < 0.05. All data were analyzed using GraphPad Prism 6 software.

### Reporting summary

Further information on research design is available in the Nature Research Reporting Summary linked to this article.

## Supplementary information

Supplementary Information

Description of Additional Supplementary Files

Supplementary Data 1

Reporting Summary

## Data Availability

All relevant data are available from the authors upon request and the corresponding author will be responsible for replying to the request. Source data underlying plots shown in figures are provided in Supplementary Data 1. Full blots are shown in Supplementary Fig. 7 of Supplementary Information.
