# Supplementary material for: Influenza virus neuraminidase regulates host CD8+ T-cell response in mice
Source: Commun Biol. 2020 Dec 8;3:748. doi: 10.1038/s42003-020-01486-z (PMC7722854; doi:10.1038/s42003-020-01486-z)

Supplementary Fig 1.

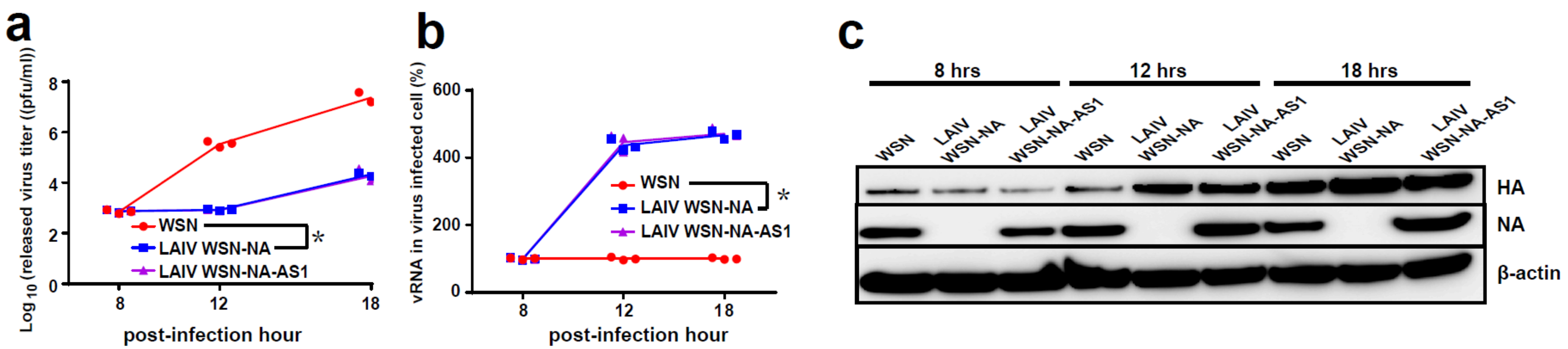

Supplementary Fig. 1 Characterization of NA-defective IAV in MDCK cells. MDCK cells were infected with virus at an MOI of 3. **a,** comparison of the titer of virus released from MDCK cells; **b,** comparison of intracellular vRNA by Q-PCR; **c,** viral proteins expressed in MDCK cells were analyzed by western blot. The filter was probed with anti-HA, anti-NA and anti-β-actin antibodies. **c, d,** Mean ± SD of three independent experiments. **P* < 0.001.

Supplementary Fig. 2

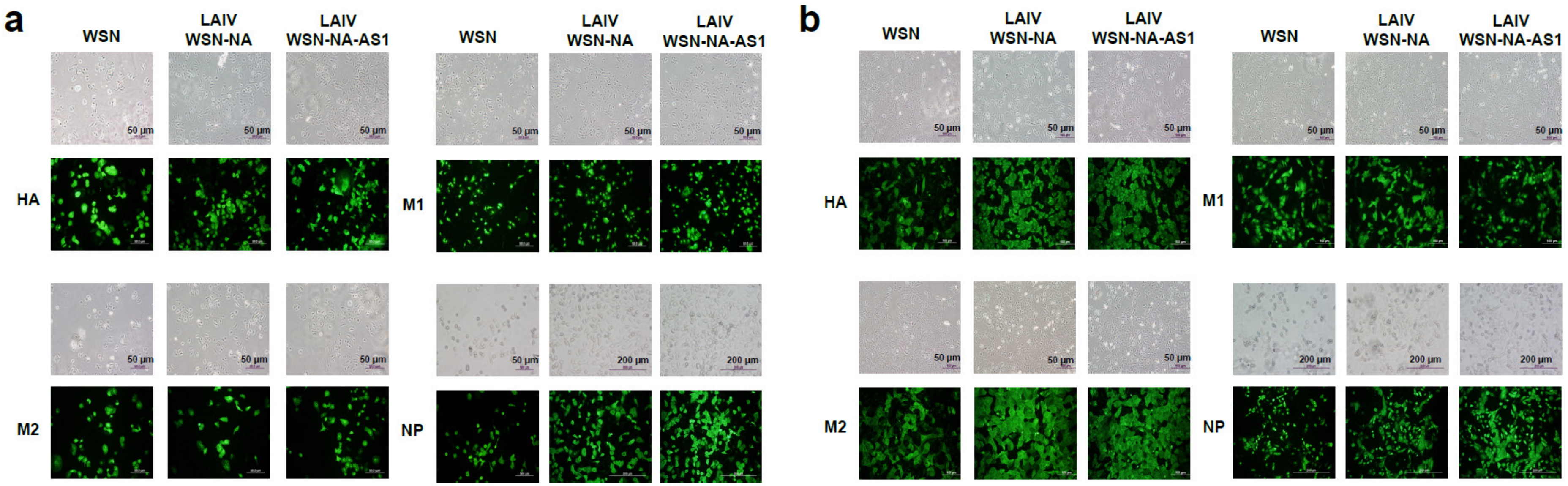

Supplementary Fig. 2 Distribution of IAV proteins in A549 and MDCK cells. After A549 **a,** and MDCK **b**, cells infected by WSN, LAIV WSN-NA or WSN-NA-AS1 at an MOI of 3 at 24 h post-infection, the IAV-infected cells were immunostained with anti-HA, anti-M2, anti-M1 and anti-NP antibodies. The top panels (without green fluorescence) showed cell morphology, and the bottom panel with green fluorescence indicated the viral proteins immunostained by the antibodies described above.

Supplementary Fig. 3

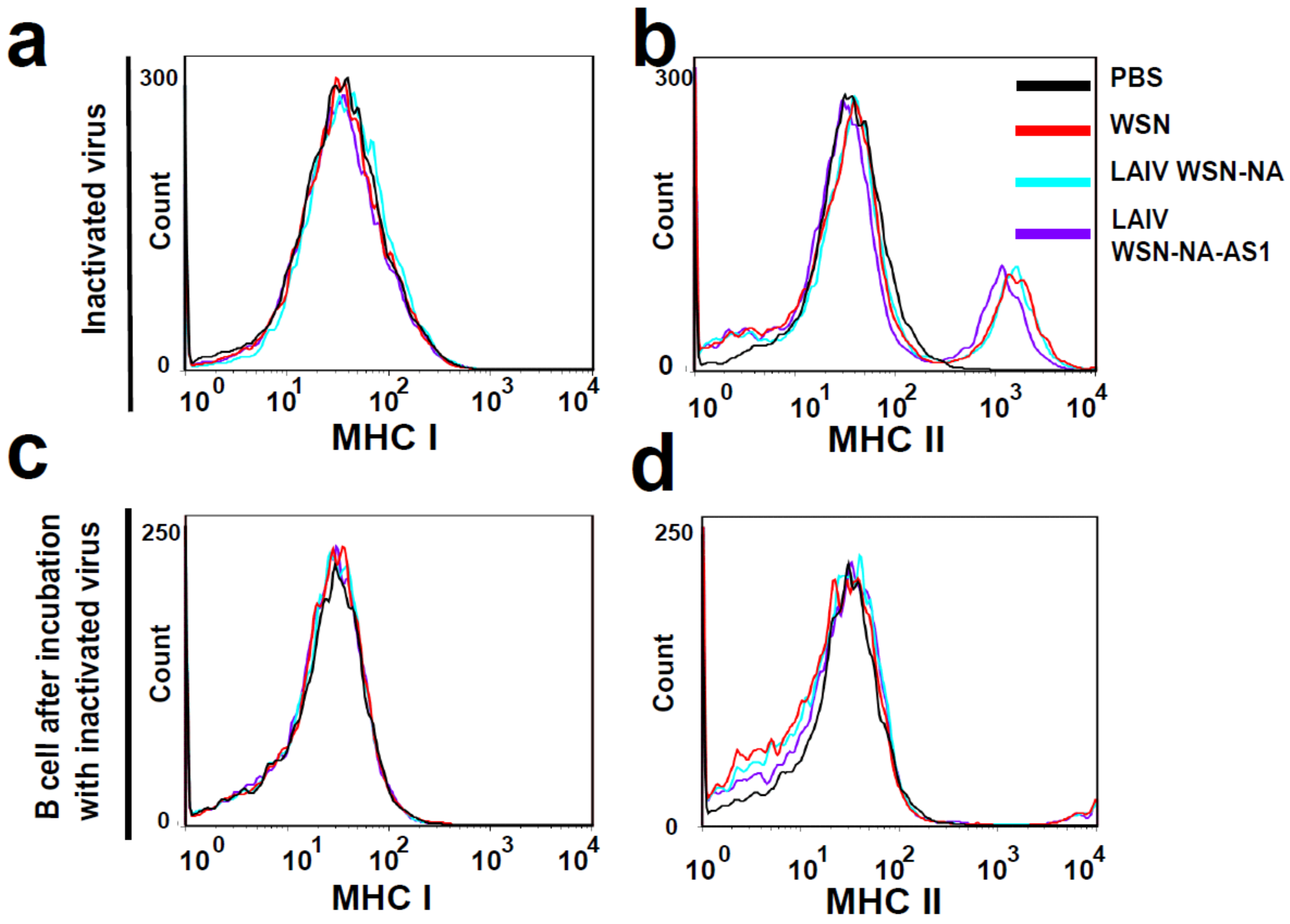

Supplementary Fig. 3 Relationship between DCs and WT WSN and LAIV WSN variants. Representative flow cytometry histograms of MHC I (**a**) and MHC II (**b**) expression on DCs after incubation with wt WSN and LAIV WSN variants. MHC I (**c**) and MHC II (**d**) expressed on DCs after incubation with the cells treated with inactivated IAV. Data are representative of two experiments.

Supplementary Fig. 4

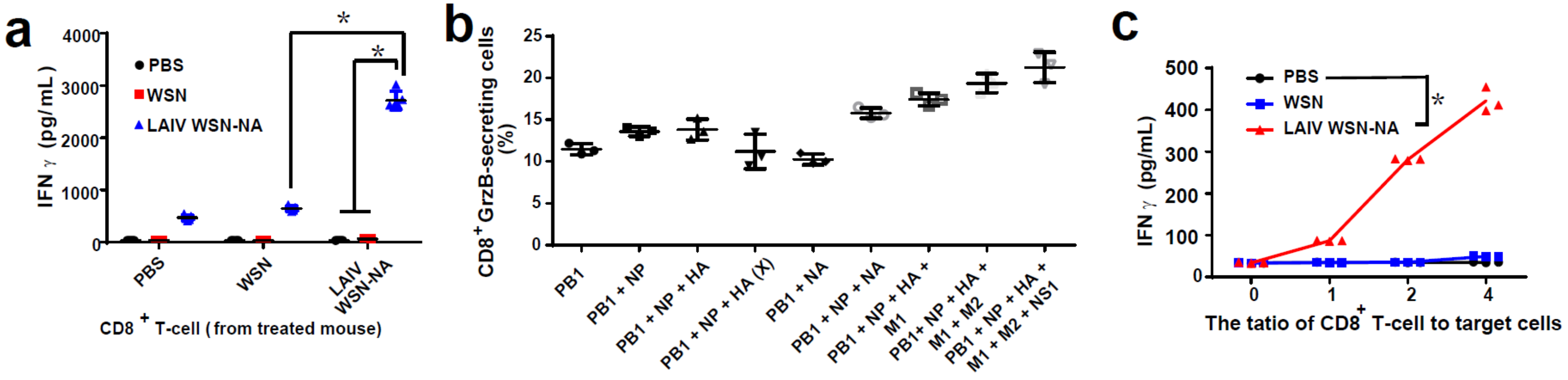

Supplementary Fig. 4 Characterization of IAV-specific CD8⁺ T cells. a, IFNγ production of CD8⁺ T cells from mice treated with DCs that were co-cultured with different virus infected cells. **b,** Representative flow cytometry histograms of GrzB expression after the IAV-specific CD8⁺ T cells were incubated with different epitope regions of viral proteins that were shown in the figure. Following Fig. 3n, measurement of GrzB secreting IAV-specific CD8⁺ T cells with addition of more different viral peptides by flow cytometry. HA (X) indicated the peptide (KESTQKAIDGVTNKVNS) of HA that did not exist in WSN. PB1: VSDGGPNLY; NP: CTELKLSDY; HA: RGLFGAIAGFIE; M1: GILGFVFTL; M2: VETPIRNEW; NS1: AIMDKNIIL. **c,** Cytotoxicity of IAV-specific CD8⁺ T cells. The secreted IFNγ was measured by ELISA. **a,** Data are representative of two similar experiments. **a, c, d,** Mean ± SD for three independent experiments. Data are representative of three

experiments.

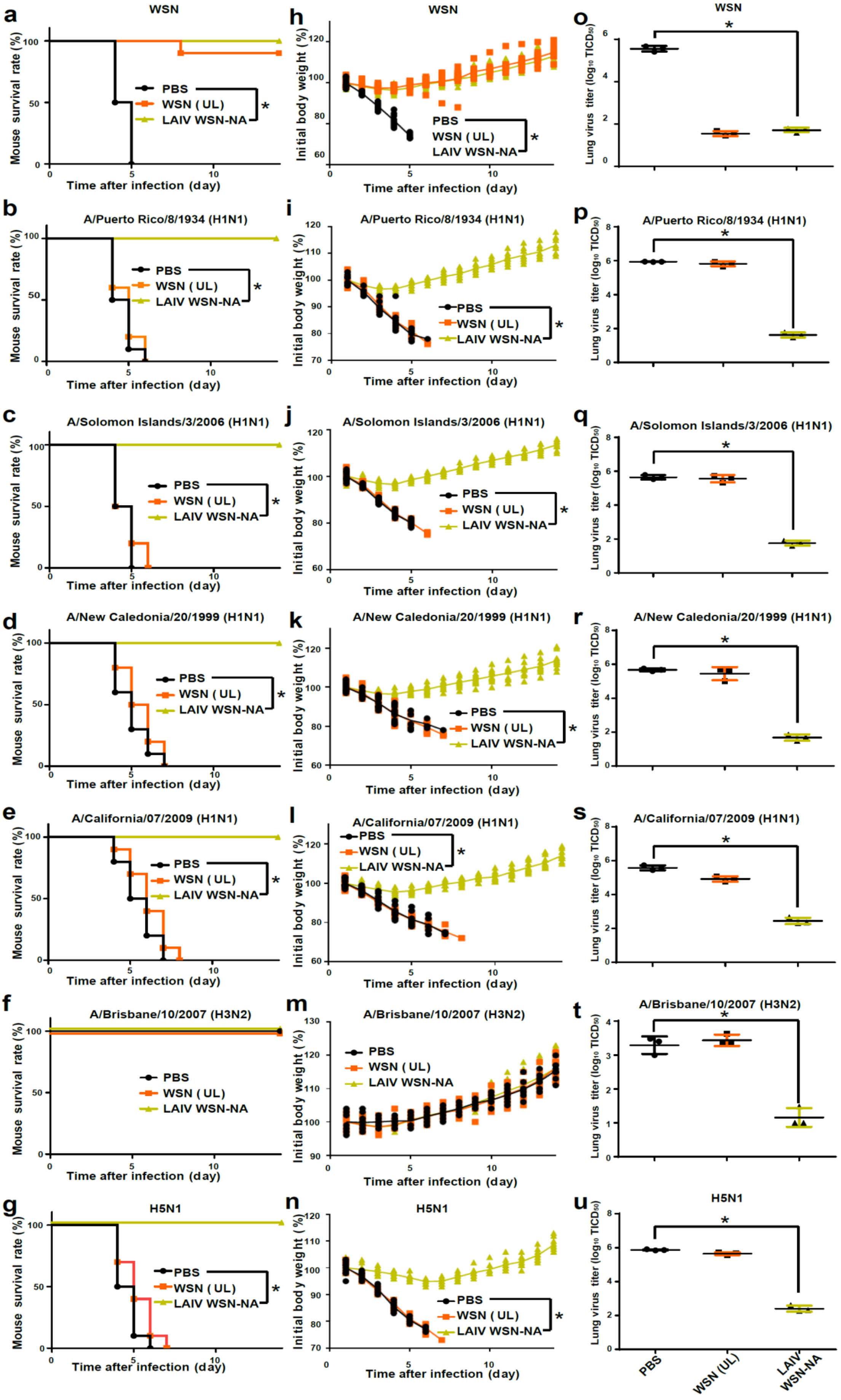
a
WSN
Mouse survival rate (%)
Time after infection (day)
PBS
WSN (UL)
LAIV WSN-NA
*
b
A/Puerto Rico/8/1934 (H1N1)
c
A/Solomon Islands/3/2006 (H1N1)
d
A/New Caledonia/20/1999 (H1N1)
e
A/California/07/2009 (H1N1)
f
A/Brisbane/10/2007 (H3N2)
g
H5N1
h
i
j
k
l
m
n
Initial body weight (%)
o
p
q
r
s
t
u
Lung virus titer (log10 TICD50)
PBS
WSN (UL)
LAIV WSN-NA

Supplementary Fig 5. The cross-protection ability of mice immunized with LAIV WSN-NA. Analysis of the survival rate (**a**-**g**), body weight (**h**-**n**) and IAV replication kinetics (**o**-**u**) in the lungs of mice immunized with WSN (UL), LAIV WSN-NA and PBS on day-4 post-infection after challenge with different strains of IAV as shown in the figure. **a**-**g,** Ten independent experiments are shown. **h-n,** Mean ± SD for ten independent experiments. **o-u,** Mean ± SD for three independent experiments. $*P < 0.001$.

Supplementary Fig. 6

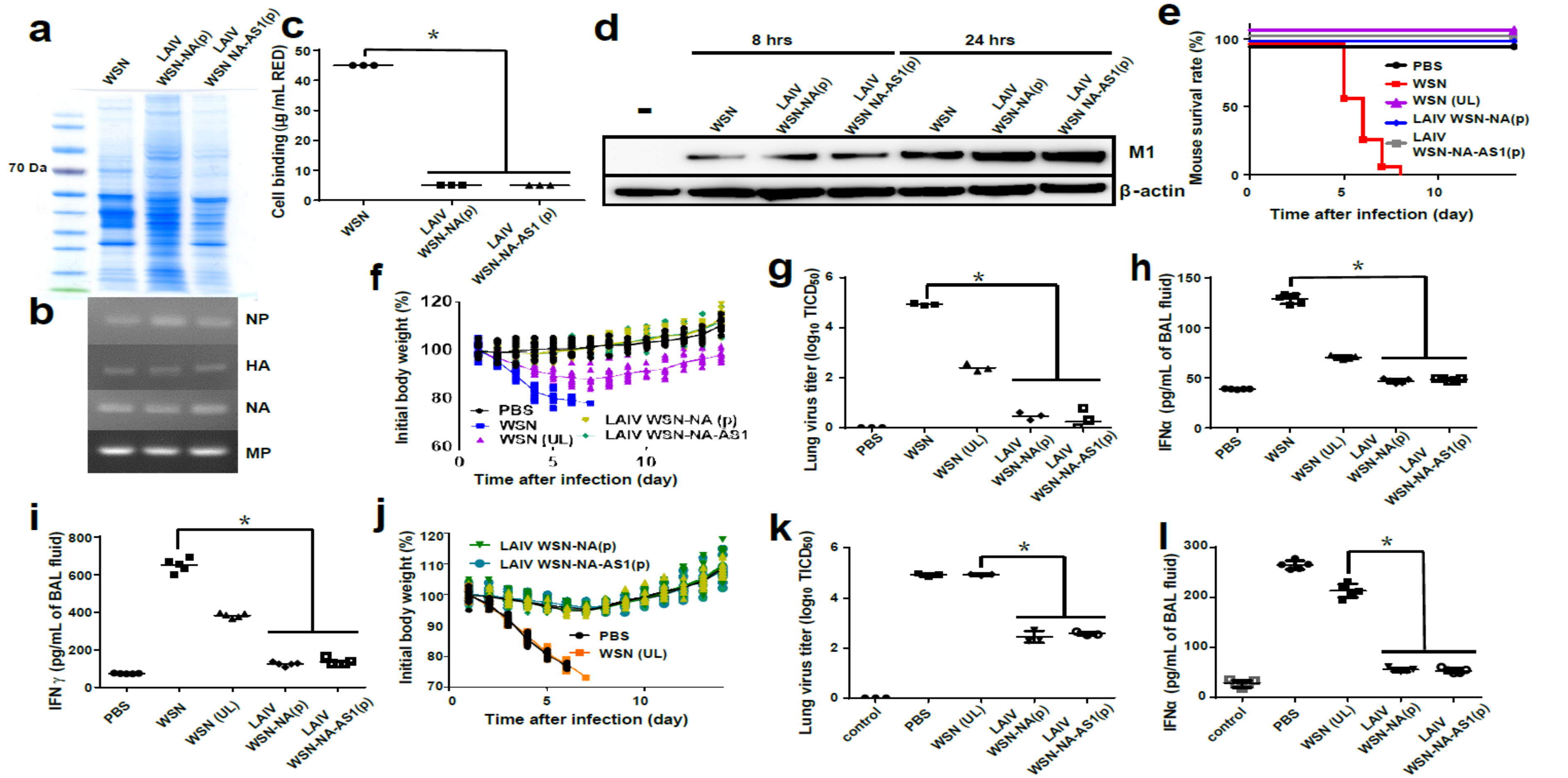

Supplementary Fig. 6 Characterization of the viral particles isolated from MDCK cells infected by NA-defective virus. a, Instant blue staining of infection particles from MDCK cells. **b,** vRNA in the infection particles from MDCK cells. **c,** Measurement of the binding avidity of infection particles from MDCK cells by cell binding assay. **d,** After A549 cells were infected by NA-defective WSN, the viral protein expression was analyzed by western blot. The filter was probed with anti-M1 and anti-β-actin antibodies. After mice were infected with$1x10^6$ PFU of WSN, WSN (UL) or the viral particles (LAIV WSN-NA(p), WSN-NA-AS1(p)) produced from MDCK cells, the survival rate (**e**) and body weight (**f**) were recorded for 14 days. **g,** The virus replication kinetics in the lung of virus treated mice on day-4 post-infection. Measurement of IFNα (**h**) and IFNγ (**i**) from the BAL fluid of infected virus at day-4 post-infection. **J,** Analysis of the body weight of WSN, LAIV WSN-NA(p), WSN-NA-AS1(p) or PBS treated mice after challenge with H5N1. **k,** H5N1 virus replication kinetics in the lung of treated mice on day-4 post-infection. **l,** IFNα from BAL fluid during H5N1 infection was measured at day-4 post-infection. The control indicated the mice without IAV infection. **c, g, h, k, l,** Mean ± SD for three independent experiments. **e,** Ten independent experiments are shown. **f, j,** Mean ± SD for ten independent experiments. *$P < 0.001$.

Supplementary. Fig 7

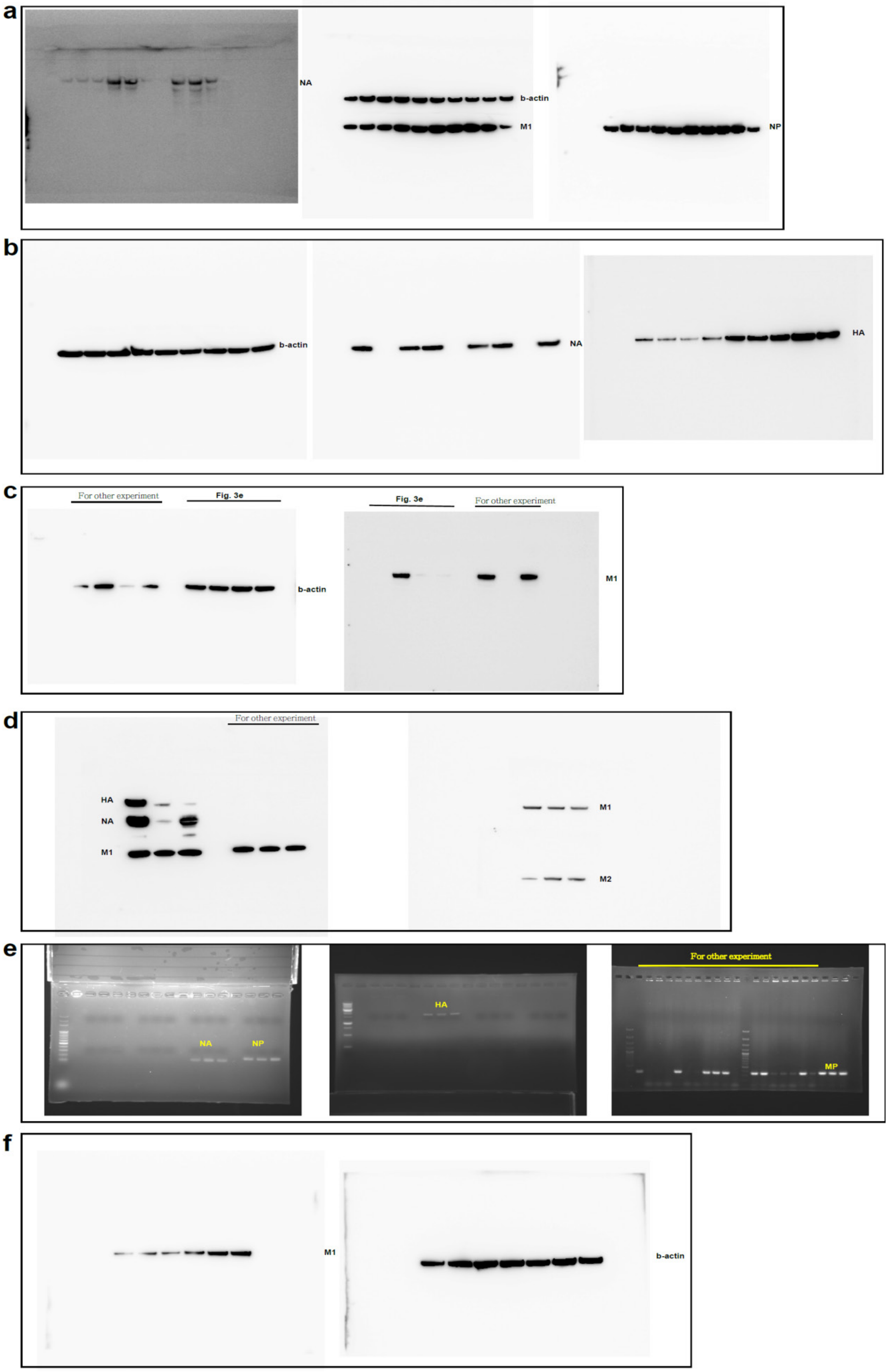

Supplement: Supplementary file 1 — Supplementary Information [file 42003_2020_1486_MOESM1_ESM.pdf]
