# Supplementary material for: Influenza virus neuraminidase regulates host CD8+ T-cell response in mice
Source: Commun Biol. 2020 Dec 8;3:748. doi: 10.1038/s42003-020-01486-z (PMC7722854; doi:10.1038/s42003-020-01486-z)
Supplement: Supplementary file 2 — Description of Additional Supplementary Files [file 42003_2020_1486_MOESM2_ESM.pdf]

Description of Additional Supplementary Files

File Name: Supplementary Data 1
Description: Raw data were used to construct graphs of figures as shown in this paper.
