# Supplementary material for: Influenza virus neuraminidase regulates host CD8+ T-cell response in mice
Source: Commun Biol. 2020 Dec 8;3:748. doi: 10.1038/s42003-020-01486-z (PMC7722854; doi:10.1038/s42003-020-01486-z)
Supplement: Supplementary file 4 — Reporting Summary [file 42003_2020_1486_MOESM4_ESM.pdf]

nature research

Corresponding author(s): Chi-Huey Wong. COMMSBIO-20-1141B

Last updated by author(s): Oct 22, 2020

Reporting Summary

Nature Research wishes to improve the reproducibility of the work that we publish. This form provides structure for consistency and transparency in reporting. For further information on Nature Research policies, see our Editorial Policies and the Editorial Policy Checklist.

Statistics

For all statistical analyses, confirm that the following items are present in the figure legend, table legend, main text, or Methods section.

n/a	Confirmed	
☐	☒	The exact sample size (*n*) for each experimental group/condition, given as a discrete number and unit of measurement
☐	☒	A statement on whether measurements were taken from distinct samples or whether the same sample was measured repeatedly
☐	☒	The statistical test(s) used AND whether they are one- or two-sided<br>*Only common tests should be described solely by name; describe more complex techniques in the Methods section.*
☒	☐	A description of all covariates tested
☒	☐	A description of any assumptions or corrections, such as tests of normality and adjustment for multiple comparisons
☒	☐	A full description of the statistical parameters including central tendency (e.g. means) or other basic estimates (e.g. regression coefficient) AND variation (e.g. standard deviation) or associated estimates of uncertainty (e.g. confidence intervals)
☐	☒	For null hypothesis testing, the test statistic (e.g. *F*, *t*, *r*) with confidence intervals, effect sizes, degrees of freedom and *P* value noted<br>*Give P values as exact values whenever suitable.*
☒	☐	For Bayesian analysis, information on the choice of priors and Markov chain Monte Carlo settings
☐	☒	For hierarchical and complex designs, identification of the appropriate level for tests and full reporting of outcomes
☒	☐	Estimates of effect sizes (e.g. Cohen's *d*, Pearson's *r*), indicating how they were calculated

Our web collection on statistics for biologists contains articles on many of the points above.

Software and code

Policy information about availability of computer code

Data collection	no software used
Data analysis	Prism 6 was also used to analyze data.

For manuscripts utilizing custom algorithms or software that are central to the research but not yet described in published literature, software must be made available to editors and reviewers. We strongly encourage code deposition in a community repository (e.g. GitHub). See the Nature Research guidelines for submitting code & software for further information.

Data

Policy information about availability of data

All manuscripts must include a data availability statement. This statement should provide the following information, where applicable:

- Accession codes, unique identifiers, or web links for publicly available datasets
- A list of figures that have associated raw data
- A description of any restrictions on data availability

Generated date are available as Figure 1-4 and Supplementary information

Field-specific reporting

Life sciences study design

All studies must disclose on these points even when the disclosure is negative.

Sample size	group of 10 mice were studied in order to achieve statistically significant information according to nQuery prediction.
Data exclusions	no data was excluded from the analysis.
Replication	All attempts at at replication were successful.
Randomization	The female 4–6-week-old BALB/c mice were studied because it was the standard models for influenza A virus study.
Blinding	We used blinded test for all animal studies.

Reporting for specific materials, systems and methods

We require information from authors about some types of materials, experimental systems and methods used in many studies. Here, indicate whether each material, system or method listed is relevant to your study. If you are not sure if a list item applies to your research, read the appropriate section before selecting a response.

Materials & experimental systems

n/a	Involved in the study
☐	☒ Antibodies
☐	☒ Eukaryotic cell lines
☒	☐ Palaeontology and archaeology
☐	☒ Animals and other organisms
☒	☐ Human research participants
☒	☐ Clinical data
☒	☐ Dual use research of concern

Methods

n/a	Involved in the study
☒	☐ ChIP-seq
☐	☒ Flow cytometry
☒	☐ MRI-based neuroimaging

Antibodies

Antibodies used	Mouse monoclonal anti-HA, anti-NA, anti-NP, anti-M2 and anti-β-actin. Rabbit monoclonal anti-MHCII and anti-MHC I. Rabbit polyclonal anti-granzyme B antibody. Goat polyclonal anti-M1 antibody.
Validation	All commercial antibodies were validated for specificity by companies and us via western blot.

Eukaryotic cell lines

Policy information about cell lines

Cell line source(s)	Madin-Darby canine kidney cells (MDCK), human embryonic kidney cells (HEK293T) and A549 human adenocarcinomic alveolar basal epithelial cells were purchased from ATCC.
Authentication	None of the cell lines used were authenticated.
Mycoplasma contamination	All cell lines tested negative for mycoplasma contamination.
Commonly misidentified lines (See ICLAC register)	No misidentified cell lines.

Animals and other organisms

Policy information about studies involving animals; ARRIVE guidelines recommended for reporting animal research

Laboratory animals	The female 4–6-week-old BALB/c mice were used in this study.
Wild animals	This study did not involve wild animals.
Field-collected samples	The study did not involve samples collect from the field.
Ethics oversight	We followed the guidance from Experimental Animal Facilities in Academia Sinica, Taiwan.

Note that full information on the approval of the study protocol must also be provided in the manuscript.

Flow Cytometry

Plots

Confirm that:

- [x] The axis labels state the marker and fluorochrome used (e.g. CD4-FITC).
- [x] The axis scales are clearly visible. Include numbers along axes only for bottom left plot of group (a 'group' is an analysis of identical markers).
- [x] All plots are contour plots with outliers or pseudocolor plots.
- [x] A numerical value for number of cells or percentage (with statistics) is provided.

Methodology

Sample preparation	CD8 T cell and dendritic cells were isolated from mice by using M-pluriBead Cell Separation kit (pluriSelect).
Instrument	The anti-granzyme B, histocompatibility complex class I and class II were used, and models number was 30000.
Software	FCS Express 3.0 software
Cell population abundance	Cells were isolated by using M-pluriBead Cell Separation kit (accroding to the specific markers on the target cells). After isolated, the purity analysis of cell were confirmed by staining with specific markers and following flow (To see any extra signals). The pure cells were stained with target antibodies or 5(6)-carboxyfluorescein diacetate N-succinimidyl ester and go to the next steps as described in the manuscript .
Gating strategy	Negative control (unstained) and positive cells (antibody binding cells)were used to establish gates for each cell type. Gates were drawn to collect cells expressing either positive and negative.

- [x] Tick this box to confirm that a figure exemplifying the gating strategy is provided in the Supplementary Information.
